# In silico identification of phytoconstituents from *Capparis sepiaria* as interleukin-1 inhibitors for rheumatoid arthritis: molecular docking, ADMET profiling, and molecular dynamics simulation

**DOI:** 10.1007/s40203-025-00386-6

**Published:** 2025-07-22

**Authors:** Darius R. Martin, Antoinette Ajmal, Mervin Meyer, Abram M. Madiehe

**Affiliations:** 1 Nanobiotechnology Research Group, Department of Biotechnology, Faculty of Natural Sciences, University of the Western Cape, Bellville, South Africa; 2 Department of Science, Technology and Innovation/TIA Nanotechnology Platform, Department of Biotechnology, Faculty of Natural Sciences, University of the Western Cape, Bellville, South Africa

**Keywords:** Molecular docking, Molecular dynamics simulations, SwissADME, Binding affinity, Anti-inflammatory

## Abstract

In this study computational methods were used to explore the anti-inflammatory properties of *Capparis* (*C.*) *sepiaria* extracts; focusing on their activity against pro-inflammatory cytokine, interleukin-1 (IL-1). Molecular docking was performed on 18 *C. sepiaria* phytoconstituents using AutoDock VinaXB. The study identified five compounds (CIDs 8122, 33934, 605626, 638072, 5363269) with high affinity for IL-1. Notably, CID 638072 demonstrated superior binding affinity compared to standard controls such as thalidomide. Pharmacokinetic and toxicity profiles were assessed using SwissADME and pkCSM which showed that all these compounds met acceptable criteria as promising anti-inflammatory agents. Molecular dynamics simulations with GROMACS (version 2019) confirmed the stability and interaction dynamics of these compounds, which support the docking results. The findings validate the traditional medicinal use of *C. sepiaria* for the treatment of inflammation, suggesting that CID 638072 holds significant potential for further development into a natural anti-inflammatory therapeutic. This research provides clues for the therapeutic applications of *C. sepiaria*, advancing the search for effective natural remedies for the treatment of inflammation. Further experimental validation is necessary to transition this study from computational predictions to clinical applications.

## Introduction

Rheumatoid arthritis (RA) is characterised as a chronic, inflammatory autoimmune disorder where the immune system erroneously targets healthy joint tissues, leading to synovial (the lining of the membranes that surround the joints) inflammation, pain, swelling, and progressive joint damage (Khalid et al. 2021). Left untreated, this can culminate in significant functional impairment and systemic symptoms such as fatigue and fever.

Globally, the prevalence of RA disorder is estimated to be between 0.24 and 1%, with a notable impact on individuals aged 25–50 (Akram et al. 2021; Singh et al. 2020). While the actual cause of RA is not known, different environmental and genetic factors are known to initiate activation of T cells, which contribute to RA by stimulating monocytes, macrophages, and synovial fibroblasts, leading to the production of cytokines such as interleukin-1 (IL-1) and Tumor Necrosis Factor α (TNF-α). These cytokines are central to the inflammation process in RA (Xu et al. 2018; Yap et al. 2018). As the overexpression of these two pro-inflammatory cytokines plays a role in tissue damage and joint inflammation, these cytokines may be used as therapeutic targets in the treatment of RA.

Currently, conventional RA therapies aim to prevent joint degeneration by targeting inflammatory mediators, thus reducing inflammation and discomfort. These therapies mainly consist of Non-Steroidal Anti-Inflammatory Drugs (NSAIDs), Disease-Modifying Antirheumatic Drugs (DMARDs), Glucocorticoids (GC), and biological therapies, such as monoclonal antbodies and cytokine inhibitors, all aimed at mitigating inflammation and preventing joint degeneration (Quan et al. 2008; Xing et al. 2020). Although treatment with NSAIDs and other chemotherapeutic agents have made considerable progress, these treatments have side effects such as osteoporosis and gastrointestinal bleeding (Salihu et al. 2018). As a result, this necessitates the need for the development of new therapeutics. Traditional plant derived medicine has contributed to the discovery of numerous modern drugs.

Medicinal plants, historically the primary source of traditional medicine, are valued by patients for their fewer side effects and suitability for long-term use compared with modern pharmaceuticals. WHO’s *Traditional Medicine Strategy* 2014–2023 stated that traditional medicine is a significant part of healthcare in many low- and middle-income countries, particularly in Africa and Asia, due to accessibility and cultural acceptance (WHO 2013). It is estimated that 80% of the World’s population still rely on medical plants for their basic healthcare needs because of their affordability and ability to treat various ailments (Kebede et al. 2021). The effectiveness of medical plants in curing various diseases is due to the different bioactive compounds they contain, which are responsible for their biological properties.

*Capparis* (*C*.) *sepiaria* is an evergreen shrub, also known in Ayurveda medicine as Himsra, which belongs to the Capparidaceae family and is well-known in South Africa for its use as a treatment for a variety of ailments (Corrigan et al. 2011), presumably due to its rich array of bioactive compounds (Quadri et al. 2023; Rajesh et al. 2009). The rich phytochemical profile of *C. sepiaria* has been described previously (Krishna 2015). Comprehensive analyses of *C. sepiaria* using gas chromatography-mass spectrometry, identified bioactive compound classes, such as fatty acids, alkaloids, flavonoids, tannins, glycosides, anthraquinones, reducing sugars, saponins, terpenoids, and carbohydrates, as well as specific compounds like oleic acid, N-methylhomopiperazine, squalene, and 3-pyridinecarboxylic acid (Krishna 2015; Rajesh et al. 2010), further suggesting the potential of *C. sepiaria* as a source of therapeutic drugs.

Crucially, toxicity assessments indicate that the plant is not toxic, supporting its applicability in therapeutic contexts (Krishna 2015). Studies have demonstrated that *C. sepiaria* exhibits numerous beneficial properties, including anti-inflammatory, antibacterial, analgesic, anticancer, hepatoprotective, antioxidant, and antidiabetic effects (K et al. 2020; Madhavan et al. 2012), and have been used traditionally to treat muscular illnesses, skin infections, inflammation, wounds, and fever (Madhavan et al. 2012). The roots of *C. sepiaria* have been specifically investigated for their phytochemical and pharmacological properties, highlighting their potential in treating conditions like RA (Chaudhari et al. 2004; Jalebawi and Chyad 2022). Additionally, studies on the antibacterial activity of *C. sepiaria* leaves and fruits further emphasise its therapeutic versatility (Kalpana and Prakash 2015).

The use of *C. sepiaria* in traditional medicine for wound healing and inflammation implies that this plant may contain bioactive compounds that can target the pro-inflammatory cytokines implicated in RA. Its antibacterial effects against enteric pathogens and antioxidant potency also underscores its potential medicinal potential (Sundaram et al. 2015). The use of in silico approaches to identify novel inhibitors of cytokines such as TNF-α and IL-6 are emerging as promising tools to identify therapeutic agents for the treatment of RA (Aldossari et al. 2023). Several studies have identified TNF-α inhibitors through network pharmacology and molecular docking, highlighting the promise of computational methods in drug discovery (Bai et al. 2021; Zia et al. 2020). Additionally, predictive models like TNF i pred aim to classify potential TNF-α inhibitors, advancing our understanding of therapeutic targets (Prabha et al. 2024).

To date, no studies have specifically investigated the potential of the phytochemicals from *C. sepiaria* as IL-1 inhibitors. Therefore, the current study aims to use an in silico approach to screen the phytochemicals of *C. sepiaria* to identify compounds that may act as IL-1 ligands.

## Materials and methods

### Protein and ligand selection and preparation

The 3D structure of IL-1 (with identifier PDB ID: 2KKI) was retrieved from the Protein Data (PDB, https://www.rcsb.org/) using the method described by Mohan and Yu (2011). The 2KKI structure was selected based on its structural accuracy as determined by: (1) its high resolution (2.1 Å), (2) well-characterised binding site relevant to RA inflammation, (3) prior use in docking studies, and (4) compatibility with docking and simulation tools (Mohan and Yu 2011). The chain of the IL-1 (2KKI) was prepared via the MGL Autodock tools (ADT) (https://ccsb.scripps.edu/mgltools/). The preparation included geometry optimization to correct structural anomalies, energy minimisation to achieve a stable conformation, and the addition of polar hydrogens and Gasteiger charges to mimic physiological conditions. Water was removed, missing residues were added, and partial charges were assigned to atoms as per the established protocol (Sanner 1999). The processed protein structure was then saved in pdbqt format, the standard for AutoDock docking simulations.

For the ligand selection process, sixteen phytoconstituents from *C. sepiaria* were selected based on phytochemical analysis by Rajesh et al., (2010). Additionally, the compounds used as controls (thalidomide, α-Amyrin and Lupeol) in this study are known to interact with IL-1 as demonstrated by Yende et al., (2021). These ligands were also prepared using MGL ADT, where Gasteiger charges were added to reflect the electronic properties of the molecules correctly, and the structures were converted to pdbqt format.

Preparation of the protein and ligands ensured precise docking simulations, aiming to uncover potential interactions between the phytoconstituents and the target in the context of RA therapy.

### Molecular docking calculations

All selected phytoconstituents were employed as ligands, while the protein structure of 2KKI was utilised as receptor protein targeting IL-1.

To facilitate the docking process, the inhibitor binding sites of 2KKI (Chang et al. 2010; Mohan and Yu 2011) were used to define the grid box, where a larger grid of 46 Å × 46 Å × 46 Å was established, centered at 23.073 Å × 4.81 Å × - −3.337 Å. These grids were crucial for directing the docking algorithm to explore potential binding sites on the protein surfaces.

Molecular docking was conducted using AutoDock VinaXB, leveraging the computational resources at the Centre for High Performance Computing (CHPC) in South Africa (Koebel et al. 2016). The results were analysed and visualised using LigPlot + version 2.2.4 (Laskowski and Swindells 2011) for 2D representations of the protein–ligand interactions, and PyMol version 2.5 (https://pymol.org/2/) for 3D visualisations. In addition, the docked complexes underwent further scrutiny using the Protein–Ligand Interaction Profiler (PLIP, https://plip-tool.biotec.tu-dresden.de/plip-web/plip/index) to confirm interaction regions/modes between ligands and the receptor. Only complexes that met the cut-off of ≥  − 6.0 kcal/mol, were selected for molecular dynamics (MD) simulations. This selection criterion was set to identify those compounds with potentially significant therapeutic relevance for further exploration in terms of stability and dynamic interactions with the target proteins.

### Absorption, distribution, metabolism, and excretion and toxicity (ADMET) studies

To ensure the viability of the selected phytoconstituents for drug development, an analysis for drug-likeness using Lipinski’s rule of five was conducted. This step is crucial to predict the tolerability of phytochemicals before being administered to humans and animal models. The pharmacokinetic profile and toxicity (ADMET) predictions of ligands were investigated using SwissADME (http://www.swissadme.ch) and pkCSM (http://biosig.unimelb.edu.au/pkcsm/prediction). SwissADME and pkCSM are web-based tools used to predict pharmacokinetic properties, especially for small molecules, using graph-based signatures. This approach was included to identify phytoconstituents with potentially unfavorable pharmacokinetic or safety profiles early in the discovery process, therefore optimising the selection for further development.

### Molecular dynamics (MD) simulations

The docked complexes were subjected to MD simulations using GROningen MOlecular Simulation (GROMACS) 2019 package (Hess et al. 2008; Pronk et al. 2013). Prior to MD simulations, the atomic coordinates were processed through the Chemistry at HARvard Macromolecular Mechanics (CHARMM)-GUI server, where CHARMM36M force field (FF) was used to define the complex topologies (Brooks et al. 2009; Jo et al. 2008; Lee et al. 2016). These complexes were then placed within a 10 Å rectangular cubic box, with each dimension measuring 89 Å, maintaining orthogonal angles of 90.0° for all axes.

For simulation under Periodic Boundary Conditions (PBCs), the necessary grid parameters for the Particle Mesh Ewald (PME), the Fast Fourier Transform (FFT) were generated automatically (Brooks et al. 2009; Jo et al. 2008; Lee et al. 2016), ensuring the grid extended beyond the simulation box.

The equilibration of the systems was carried out in two phases, for 125 ps (125,000 steps) and 250 ps (250,000 steps) using NVT and NPT ensembles, at 310 K temperature, respectively. In the NVT ensemble, the number of particles (N), volume (V) and temperature (T) were all kept fixed throughout the simulation. On the other hand, the NPT ensemble was performed to equilibrate pressure (P) and density of the systems at the same temperature.

The complexes were immersed in a water box, filled with Simple Point Charge (SPC) water molecules and were neutralised with potassium (K^+^) and chloride (Cl^−^) counterions, at a default concentration of 0.15 M, using the Monte-Carlo method for ion placement (Brooks et al. 2009; Jo et al. 2008; Lee et al. 2016). The number of ions were automatically determined by the ion-accessible volume (*V*), the total charge of the systems (*Q*_*sys*_) and the valence of the positive ion (Z_+_), respectively.

Subsequently, the systems were energy minimised for 50 ps (50,000 steps) with the steepest descent algorithm to remove any initial steric clashes between atoms from the starting structures until a tolerance of 2500 kJ/mol was reached. Energy minimisations were followed by 1 ns equilibration under NVT and NPT ensembles with position restraints applied to the protein to stabilise the solvent environment.

Finally, each system was simulated for 500 ns at a reduced temperature of 278 K temperature and 1 bar pressure, with all restraints removed. Bond constraints were managed using the LINear Constraint Solver (LINCS) algorithm (Hess et al. 2008), and long-range electrostatic interactions were computed via the PME algorithm (Darden et al. 1993), with a cut-off distance of 1.2 nm for short-range electrostatic interactions.

This method ensured a thorough examination of the complex dynamics, balancing computational efficiency with accuracy.

#### Root mean square deviation (RMSD), radius of gyration (RyG), root mean square fluctuation (RMSF) and hydrogen (H) bonds calculations.

The MD simulations were performed under PBCs in all directions, with subsequent analysis conducted using various GROMACS tools. The gmx_rmsd was used to calculate the Root Mean Square Deviation (RMSD) of the protein backbone in complex with phytoconstituents, offering insights into structural stability. The gmx_gyr was employed to calculate the radius of gyration (RyG), an indicator of protein compactness or unfolding. The gmx_rmsf tool measured the root mean square fluctuation (RMSF) of protein residues, highlighting regions of high flexibility such as loops or termini. Lastly, gmx_hbonds evaluated the hydrogen bond formations between the protein and phytoconstituents over the selected period of the simulation, providing data on the stability and specificity of the interactions (Martin et al. 2023).

These analyses provide a comprehensive view of dynamic behavior, structural integrity, and molecular interaction specifics within the protein- phytoconstituents complexes.

#### Non-bonded interaction energy calculation

The strength of the association between phytoconstituents and proteins was quantified by calculating the total average non-bonded interaction energy across the 200 ns simulation time. This energy, denoted as E^int^, is determined by the equation:

$$ {\text{E}}^{{{\text{int}}}} = {\text{ L}} - {\text{J }} + {\text{ Coul}} $$

Here, E^int^ represents the average energy in Kcal/mol obtained from the energy file, L-J signifies the short-range Lennard–Jones interaction energy, and Coul is the Coulomb interaction energy. The gmx_energy tool within GROMACS software was used to derive these average values for the short-range L-J and Coul interaction energies within the molecular complexes, respectively.

#### Binding free energy calculations

The binding free energies of the complexes were calculated using the gmx_MMPBSA tool (version 1.4.3) from GROMACS, implementing the AMBER MMPBSA.py script at version 16.0 (Paissoni et al. 2015; Valdés-Tresanco et al. 2021). The molecular mechanics energies with Posson-Boltzmann and solvent-accessible surface area continuum solvation methods (MMPBSA) tool offers a c comprehensive strategy for assessing the binding free energies of protein–ligand complexes (Gilson and Zhou 2007; Huo et al. 2002; Moreira et al. 2010; Reddy et al. 2014). The binding free energy, denoted as ΔG, is calculated using the equation:

$$ \Delta {\text{G }} = \, \Delta {\text{H }} - {\text{T}}\Delta {\text{S}} $$

Here, ΔG signifies the change in binding free energy, ΔH is the change in enthalpy, T is the absolute temperature, and ΔS is the change in entropy (Bradshaw et al. 2011; Brown and Muchmore 2009; Gilson and Zhou 2007; Huo et al. 2002; Massova and Kollman 1999; Moreira et al. 2010; Reddy et al. 2014).

This thermodynamic relationship provides crucial insights into the molecular interactions that drive complex formation between proteins and ligands. The change in entropy (ΔS) in MM-PBSA is evaluated through Normal Mode Analysis (NMA), which examines the entropy by considering the harmonic motions of the atoms. The process involves minimising the structures from MD simulations to remove high-energy conformations that do not represent the typical energy landscape of the system. The vibrational frequencies are then computed for the complex, the receptor, and the ligand in their bound and unbound states. From these vibrational frequencies, the entropy is then calculated using the statistical mechanics principles with the formula;

$$ \Delta {\text{S }} = {\text{ S}}_{{{\text{complex}}}} - \, \left( {{\text{S}}_{{{\text{receptor}}}} + {\text{ S}}_{{{\text{ligand}}}} } \right) $$

#### Principal component analysis (PCA)

Principal Component Analysis (PCA) is a statistical method that simplifies complex data sets by separating biologically significant protein domain movements from less significant localised atomic motions (David and Jacobs 2014). As a linear transformation method, PCA identifies key movements within the data using a covariance matrix derived from atomic coordinates, which represent the degrees of freedom of protein residues (David and Jacobs 2014). This approach transforms the data into a new coordinate system, where the most significant variance (determined by the scalar projection of the data) is aligned with the first principal component, and the second most significant variance is aligned with the second coordinate (Ayaz 2003).

A covariance matrix was generated using gmx_mpi covar, to examine how atomic positions deviate from their mean in relation to one another. The eigenvectors and eigenvalues of the covariance matrix were then computed, to identify the principal components. The eigenvectors (of the covariance matrix) were used to indicate the directions of the most significant variance, while the eigenvalues (which are the coefficients associated with eigenvectors) were used to indicate the amount of variance each principal component accounted for.

Finally, a 2D graph was created to visualise the distribution of variance along the two main principal component axes. This provided insights into the differences in protein movements throughout the simulation landscape.

The eigenvalues (coefficients attached to the eigenvectors) were obtained from the eigenvalue.xvg files. The PCA percentage (%) was calculated using the following formula for the first two eigenvalues:

$$ \left( {{\text{value no}}.{ 1}/{\text{sum of five}}} \right) \, *{ 1}00 \, = { 1}^{{{\text{st}}}} {\text{eigenvalue }}\% $$

$$ \left( {{\text{value no}}.{ 2}/{\text{sum of five}}} \right) \, *{ 1}00 \, = { 2}^{{{\text{nd}}}} {\text{eigenvalue }}\% $$

$$ \begin{gathered} {\text{then the sum of the 1}}^{{{\text{st}}}} {\text{eigenvalue }}\% \hfill \\ \;\;\;\;{\text{and 2}}^{{{\text{nd}}}} {\text{eigenvalue }}\% {\mkern 1mu} = {\mkern 1mu} \ge 50\% \hfill \\ \end{gathered} $$

## Results

### Protein and ligand selection and preparation

**Fig. 1:**
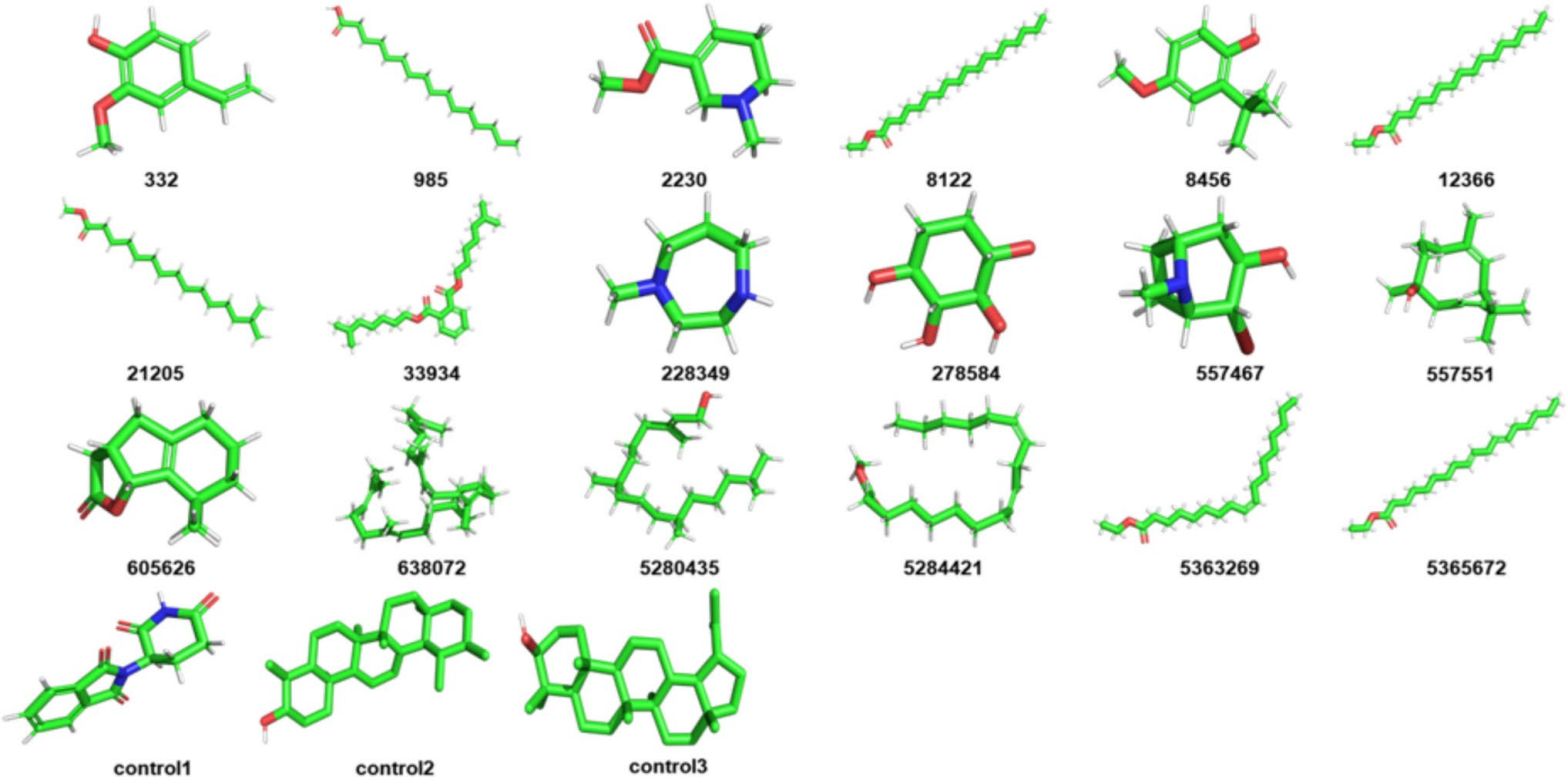
Phytoconstituents identified in *C. sepiaria* extracts (Rajesh et al. 2010; Yende et al. 2021). Compounds with identification (CID) 332: 2-Methoxy-4-vinylphenol, CID 985: Palmitic Acid, CID 2230: Arecoline, CID 8122: Ethyl Stearate, CID 8456: 3-tert-Butyl-4-hydroxyanisole, CID 12366: Ethyl Palmitate, CID 21205: Methyl 14-methylpentadecanoate, CID 33934: Diisooctyl Phthalate, CID 228349: N-Methylhomopiperazine, CID 278584: 1,2,3,4-Cyclohexanetetrol, CID 557467: 2-Bromo-8-methyl-8-azabicyclo[3.2.1]octan-3-ol, CID 557551: 3,7-Cycloundecadien-1-ol, CID 605626:8,8-Dimethyl-3,3a,4,5,6,7,8,8b-octagydro-2H-indeno[1,2-b]furan-2-one, CID 638072: Squalene, CID 5280435: Phytol, 5,284,421: Methyl Linoleate, CID 5363269: Ethyl Oleate, CID 5365672: 9,12-Octadecadienoic Acid, Ethyl Ester, control 1: thalidomide, control 2: α-amyrin and control 3: lupeol. On the other hand, coloring represents the elements making up the compounds, including green: carbon atoms, red: oxygen atoms, blue: nitrogen atoms and light blue: fluorine atoms, respectively

The 2KKI protein receptor and the ligands were successfully extracted from RCSB PDB and PubChem, which were then prepared and set ready for docking analysis. The selected ligands from *C. sepiaria* are shown in Fig. 1.

### Molecular docking calculations

**Fig. 2:**
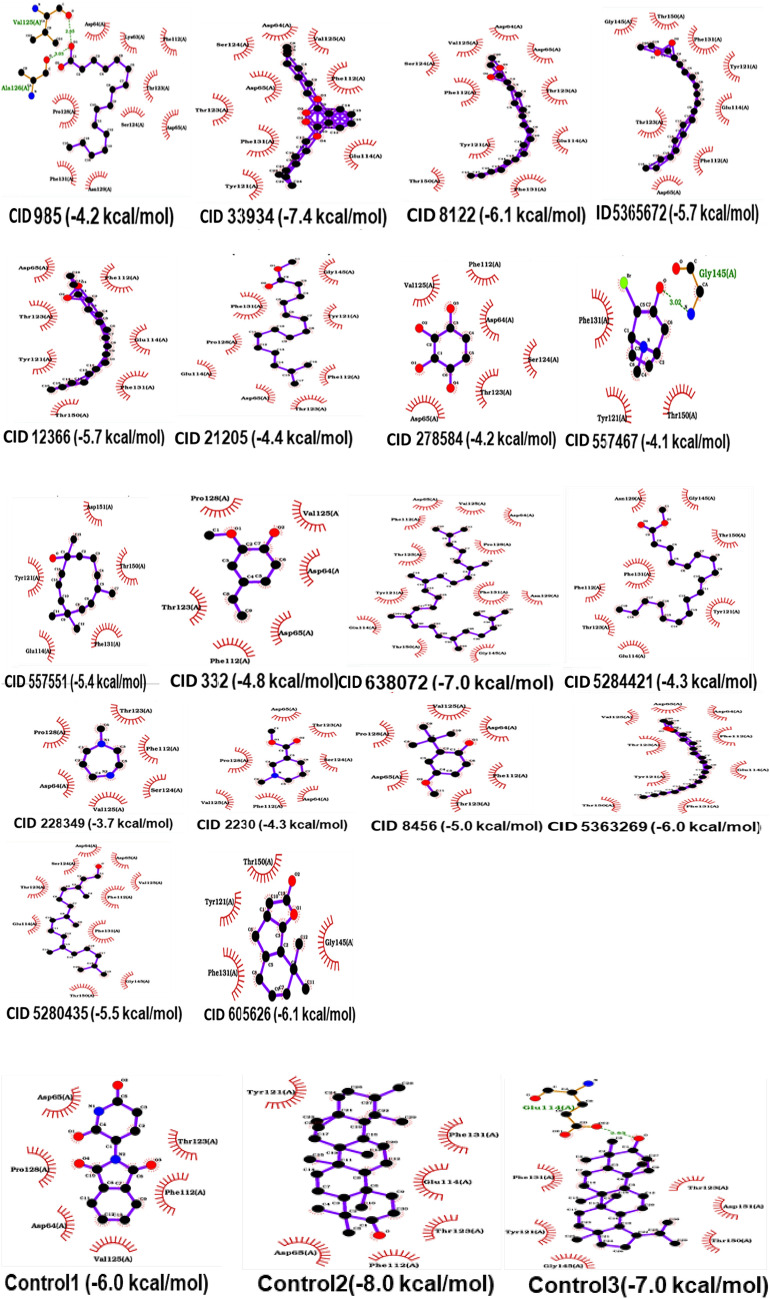
2D analysis of the docked complexes between the phytoconstituents of *C. sepiaria* and IL-1. Compounds with identification (CID) 332: 2-Methoxy-4-vinylphenol, CID 985: Palmitic Acid, CID 2230: Arecoline, CID 8122: Ethyl Stearate, CID 8456: 3-tert-Butyl-4-hydroxyanisole, CID 12366: Ethyl Palmitate, CID 21205: Methyl 14-methylpentadecanoate, CID 33934: Diisooctyl Phthalate, CID 228349: N-Methylhomopiperazine, CID 278584: 1,2,3,4-Cyclohexanetetrol, CID 557467: 2-Bromo-8-methyl-8-azabicyclo[3.2.1]octan-3-ol, CID 557551: 3,7-Cycloundecadien-1-ol, CID 605626:8,8-Dimethyl-3,3a,4,5,6,7,8,8b-octagydro-2H-indeno[1,2-b]furan-2-one, CID 638072: Squalene, CID 5280435: Phytol, CID 5284421: Methyl Linoleate, CID 5363269: Ethyl Oleate, CID 5365672: 9,12-Octadecadienoic Acid, Ethyl Ester, control1: thalidomide, control2: α-Amyrin and control3: Lupeol, respectively. Residues forming non-bonded interactions are shown as red arcs, while residues forming hydrogen bonds and the bound ligand are depicted as ball-and-stick models. Sulfur, nitrogen, oxygen, and carbon atoms are represented by orange/yellow, blue, red, and black balls. Hydrogen bonds are indicated by green dashed lines labeled, while ligand bonds are indicated by purple thick lines

Molecular docking has become an indispensable tool in the realm of drug development and discovery (Aamir et al. 2018; Meng et al. 2011). As shown in Fig. 2, the control compounds selected for this study (thalidomide, α-amyrin and lupeol), all exhibited binding energies that were equal or exceeded the set threshold of ≥  − 6.0, reflecting the generally lower binding energetics observed for IL-1. Among the compounds used as controls, control 1 (thalidomide) had a binding energy of  − 6.0 kcal/mol, which was notably weaker compared to control 2 (α-amyrin) at  − 8.0 kcal/mol and control 3 (lupeol) at  − 7.0 kcal/mol. Thalidomide, α-amyrin, and lupeol were selected as controls for docking with IL-1, as they share a common mechanism of action and binds to the same site, as illustrated in Fig. 2.

Out of the 18 phytoconstituents under scrutiny, only five achieved the requisite binding energy threshold. These compounds included CID 8122 (− 6.1 kcal/mol), CID 33934 (− 7.4 kcal/mol), CID 605626 (− 6.1 kcal/mol), CID 638072 (− 7.0 kcal/mol), and CID 5363269 (− 6.0 kcal/mol). These phytoconstituents interacted the 2KKI protein through distinct numbers of active amino acid residues: CID 8122 with 10, CID 33934 with 9, CID 605626 with 4, CID 638072 with 12, and CID 5363269 with 9.

Common active sites among these compounds included Asp64A, Asp65A, Phe112A, Glu114A, Tyr121A, Thr123A, Ser124A, Val125A, and Phe131A. However, CID 8122 uniquely interacted with Thr150, while CID 638072 and CID 5363269 did not involve Ser124. Additionally, CID 5363269 engaged three extra residues, namely Pro128, Asn129, and Gly145, while CID 605626, with its limited interaction profile, included Gly145 and Thr150.

To further elucidate the specifics of these interactions, these complexes were subjected to analysis with the PLIP web server (https://plip-tool.biotec.tu-dresden.de/plip-web/plip/index), with the findings summarised in Table 1.

**Table 1:** Modes of interactions between the selected phytoconstituents and the arthritis–related marker, 2KKI model (IL–1) using PLIP web server

Compound identification (CID)	Binding scores (kcal/mol)	Hydrophobic interactions	π–Stacking	Hydrogen bonds	Water bridges	Salt bridges	Metal complexes
332	− 4.8	ASP65A, PHE112A, PHE112A, THR123A and PRO128A	–	VAL125A	–	–	–
985	− 4.2	ASP65A, PHE112A, PHE112A, THR123A, VAL125A, PRO128A, PHE131A and PHE131A	–	VAL125A	–	–	–
2230	− 4.3	ASP65A, PHE112A, THR123A and VAL125A	–	SER124A	–	–	–
8122	− 6.1	ASP65A, PHE112A, PHE112A, PHE112A, TYR112A, THR123A, VAL125A, PHE131A and PHE131A	–	–	–	–	–
8456	− 5.0	ASP65A, PHE112A, PHE112A, PHE112A and PRO128A	–	VAL125A	–	–	–
12,366	− 5.7	PHE112A, PHE112A, TYR121A, TYR121A, PHE131A and PHE131A	–	–	–	–	–
21205	− 4.4	ASP65A, PHE112A, THR123A, PHE131A, PHE131A, PHE131A and PHE131A	–	GLY145A	–	–	–
33934	− 7.4	ASP65A, PHE112A, PHE112A, PHE112A, TYR121A, TYR121A, TYR121A, THR123A, VAL125A and PHE131A	–	–	–	–	–
228349	− 3.7	PRO128A	–	LYS63A	–	–	–
278584	− 4.2	PHE112A, PHE112A and THR123A	–	–	–	–	–
557467	− 4.1	PHE131A	–	GLY145A and GLY145A	–	–	–
557551	− 5.4	TYR121A, PHE131A and PHE131A	–	–	–	–	–
605626	− 6.1	TYR121A, PHE131A, PHE131A, PHE131A, PHE131A and THR150A	–	GLY145A	–	–	–
638072	− 7.0	ASP65A, PHE112A, PHE112A, PHE112A, TYR121A, TYR121A, TYR121A, THR123A, VAL125A, PRO128A, PHE131A, PHE131A, PHE131A and THR150A	–	–	–	–	–
5280435	− 5.5	ASP65A, PHE112A, TYR121A, THR123A, THR123A, PRO128A, PHE131A, PHE131A, PHE131A, PHE131A, PHE131A and THR150A	–	–	–	–	–
5284421	− 4.3	PHE112A, TYR121A, TYR121A, TYR121A, TYR121A, PHE131A, PHE131A and PHE131A	–	GLY145A	–	–	–
5363269	− 6.0	–	PHE209A	TYR58A, ILE59A, ILE61A, GLU86A, LYS87A, VAL88, ASN92, ARG111A and ARG111A	HIS89A, THR91A, ARG11A, LYS307A, LYS307A, ARG309A, ARG309A, ARG309A and ARG309A	LYS87A, LYS87A, ARG11A, LYS307A, ARG309A and ARG309A	ATP501A, ATP501A, HOH514A, HOH520A, HOH528A and HOH530A
5365672	− 5.7	ASP65A, PHE112A, TYR121A, TYR121A, THR123A, PHE131A, PHE131A, PHE131A, PHE131A and THR150A	–	–	–	–	–
Control 1 (5426)	− 6.0	ASP65A, PHE112A and VAL125A	PHE112A	–	–	–	–
Control 2 (α–amyrin)	− 8.0	Phe112A, Tyr121A, Tyr121A, Thr123A, Phe131A and Phe131A	–	Asp65A	–	–	–
Control 3 (Lupeol)	− 7.0	Tyr121A and Phe131A	–	Glu114A	–	–	–

–: not recorded

As shown in Table 1, all the compounds, except CID 5363269, employed hydrophobic interactions to bind to 2KKI (IL-1). Among these, CID 638072 exhibited the strongest hydrophobic interactions relative to other phytoconstituents. Furthermore, when compared to the controls, CIDs 985, 8122, 12366, 33934, 638072, 5280435, 5284421, 5363269, and 5365672 displayed stronger binding affinities.

Hydrogen bonding was also a common interaction mechanism among most compounds, including the control ligands. CID 5363269 interacted with the 2KKI protein through 1 π-stacking interaction (Phe209A), 9 hydrogen bonds (Tyr58A, Ile59A, Ile61A, Glu86A, Lys87A, Val88A, Asn92A, Arg111A, and Arg11A), 9 water bridges (His89A, Thr91A, Arg111A, Lys307A twice, Arg309A four times), 6 salt-bridges (Lys87A twice, Arg111A, Lys307A, Arg309A twice), and 6 metal complexes (ATP501A twice, HOH514A, HOH520A, HOH528A, and HOH530A).

This detailed interaction profile highlights the multifaceted binding of CID 5363269 to 2KKI protein, leveraging a wide range of interaction mechanisms.

### Absorption, distribution, metabolism, and excretion and toxicity (ADMET) studies

The pharmacokinetic and toxicological profiles of the selected phytoconstituents were thoroughly examined to gauge their clinical implications, as seen in Table 2. This analysis is fundamental to evaluate their therapeutic efficacy (Fadaka et al. 2021, 2022; Ojo et al. 2022). Understanding these properties help to predict how these compounds will behave in a clinical setting, influencing decisions on dosage, administration routes, and potential side effects.

**Table 2:** Absorption, distribution, metabolism, excretion and toxicity (ADMET) studies of the phytoconstituents of *C. sepiaria* using in silico methods

Compound identification (CID)	Compound names	Formula	MW (g/mol)	LogP	TPSA (Å2)	HB donor	HB acceptor	Acqueous solubility (Log mol/L)	Human intestinal absorption (%)	Blood–brain barrier	P-glycoprtein substrate	Total clearance [Log Ml/ (min.kg)]
332	2-Methoxy-4-vinylphenol	C9H10O2	150.17	2.04	29.5	1	2	− 1.83	94.67	0.20	No	0.25
985	Palmitic Acid	C16H32O2	256.42	5.55	37.3	1	1	− 5.64	89.44	− 0.28	No	1.76
2230	Arecoline	C8H13NO2	155.19	0.42	29.5	0	1	− 0.21	100	0.04	No	0.76
8122	Ethyl Stearate	C20H40O2	312.5	6.81	26.3	0	2	− 6.60	91.77	0.80	No	1.98
8456	3-tert-Butyl-4-hydroxyanisole	C11H16O2	180.24	2.70	29.5	1	2	− 3.06	92.68	0.18	No	0.26
12366	Ethyl Palmitate	C18H36O2	284.5	6.03	26.3	0	2	− 6.12	92.46	0.76	No	1.91
21205	Methyl 14-methylpentadecanoate	C17H34O2	270.5	5.50	26.3	0	2	− 6.00	93.31	0.75	No	1.71
33934	Diisooctyl Phthalate	C24H38O4	390.6	6.43	52.6	0	4	− 6.57	91.77	− 0.27	No	1.65
228349	N-Methylhomopiperazine	C6H14N2	114.19	− 0.09	15.3	1	2	0.53	96.41	0.03	No	0.86
278584	12,3,4-Cyclohexanetetrol	C6H12O4	148.16	− 1.78	80.9	4	4	0.11	63.08	− 0.75	No	0.52
557467	2-Bromo-8-methyl-8-azabicyclo [3.2.1] octan-3-ol	C8H14BrNO	220.11	0.98	23.5	1	2	− 0.87	95.69	− 0.03	No	0.91
557551	3,7-Cycloundecadien-1-ol	C15H26O	222.37	4.23	20.2	1	1	− 3.76	92.75	0.62	No	1.17
605,626	8,8-Dimethyl-3,3a,4,5,6,7,8,8b-octagydro-2H-indeno[1,2-b] furan-2-one	C13H18O2	206.28	2.83	26.3	0	2	− 3.22	96.71	0.55	No	0.93
638072	Squalene	C30H50	410.7	10.61	0	0	0	− 8.62	90.48	0.99	No	1.79
5280435	Phytol	C20H40O	296.5	6.36	20.2	1	1	− 7.56	91.90	0.82	No	1.69
5284421	Methyl Linoleate	C19H34O2	294.5	5.98	26.3	0	2	− 7.31	92.80	0.77	No	2.03
5363269	Ethyl Oleate	C20H38O2	310.5	6.59	26.3	0	2	− 7.56	91.87	0.79	No	2.03
5365672	9,12-Octadecadienoic Acid, Ethyl Ester	C20H36O2	308.5	6.36	26.3	0	2	− 7.49	92.38	0.78	No	2.08
Control 1 (5426)	Thalidomide	C13H10N2O4	258.23	0.09	83.55	1	4	− 2.19	69.58	− 0.31	No	1–0.06
Control 2 (73,170)	α-amyrin	C30H50O	426.7	8.02	20.23	1	1	− 6.50	94.06	0.67	Yes	0.12
Control 3 (259,846)	Lupeol	C30H50O	426.7	8.02	20							
23	1	1	− 5.86	95.78	0.73	No	0.15					

Compound identification (CID)	AMES toxicity	Max. tolerated dose [(Log mg/9 kg.d)]	Herg I inhibitor	Herg II inhibitor	Acute oral rat toxicity, LD50 (Log mg/kg bw/day)	Oral rat chronic toxicity (Log mg/kg bw/day)	Hepatotoxicity	Skin sensitisation	T. pyriformis toxicity (Log ug/L)	Minnow toxicity (Log mmol/L)
332	No	1.30	No	No	1.72	2.59	No	Yes	0.26	1.45
985	No	− 0.29	No	No	1.86	2.99	No	Yes	1.67	− 1.21
2230	Yes	0.31	No	No	2.36	2.00	Yes	Yes	− 0.29	2.41
8122	Yes	0.22	No	Yes	1.67	2.91	No	Yes	1.25	− 2.69
8456	No	0.77	No	No	1.91	2.31	No	Yes	0.96	1.05
12366	No	0.31	No	Yes	1.65	2.76	No	Yes	1.66	− 2.21
21205	No	0.15	No	No	1.60	2.73	No	Yes	1.71	− 2.03
33934	No	0.94	No	No	1.28	2.58	No	No	0.67	− 3.88
228349	No	0.89	No	No	2.22	0.88	No	Yes	− 0.28	3.12
278584	No	2.14	No	No	1.36	2.85	No	No	− 0.05	3.58
557467	Yes	0.67	No	No	2.45	0.96	Yes	Yes	− 0.22	2.39
557551	No	0.64	No	No	1.88	1.17	No	Yes	1.09	1.34
605626	No	0.34	No	No	1.88	1.92	No	Yes	0.45	1.20
638072	No	− 0.25	No	Yes	1.78	0.95	No	No	0.50	− 3.61
5280435	No	0.09	No	Yes	1.62	1.05	No	Yes	1.84	− 1.61
5284421	No	0.14	No	No	1.59	3.01	No	Yes	1.74	− 1.73
5363269	No	0.22	No	No	1.64	3.11	No	Yes	1.54	− 2.02
5365672	No	0.17	No	No	1.62	3.04	No	Yes	1.63	− 1.89
Control 1 (5426)	No	0.36	No	No	2.50	1.56	Yes	No	0.47	1.88
Control 2 (73,170)	No	− 0.57	No	Yes	2.47	0.86	No	No	0.38	− 1.31
Control 3 (259,846)	No	− 0.50	No	Yes	2.56	0.89	N0	Yes	0.32	− 1.70

The ADMET parameters were assessed through a comparison of marginal values with resultant outcomes. High Caco-2 permeability was predicted for compounds with values greater than 0.90, indicating robust absorption capabilities through the gastrointestinal tract. Intestinal absorption was considered poor for compounds with less than 30% absorption. Regarding Blood–Brain Barrier (BBB) permeability, a logBB value above 0.3 suggested that the compound could cross into the brain, while a value below − 1 indicated poor distribution. For Central Nervous System (CNS) permeability, a logPS value greater than − 2 was considered indicative of CNS penetration, with values less than − 3 suggesting no penetration. In terms of toxicity, compounds were deemed potentially harmful to *Tetrahymena pyriformis* if the logPS value exceeded − 0.5 μg/L, and high acute toxicity to minnows was predicted for compounds with a logLC50 less than − 0.3 (Pires et al. 2015; Vardhan and Sahoo 2020).

Remarkably, none of the phytoconstituents investigated showed any bystander toxicity effects, enhancing their potential for clinical use. The ADMET predictions were successfully executed in this study, providing a clear picture of how these compounds might perform in vivo, thus aiding in the strategic development of therapeutic applications.

### Molecular dynamics simulations

All phytoconstituents that met most of the criteria for oral bioavailability, as described in Section “Absorption, distribution, metabolism, and excretion and toxicity (ADMET) studies”, were subjected to MD simulations. Only thalidomide was used as a control compound for the MD simulations, while the selected phytoconstituents were CIDs 33934, 605626, 638072, 5363269, and 8122. The unbound protein structure was also employed as another control, to validate MD simulations analysis, similar to our previous study (Martin et al. 2023).

#### RMSD, RMSF, RyG, hydrogen bonds and non-bonded interaction energies

**Fig. 3:**
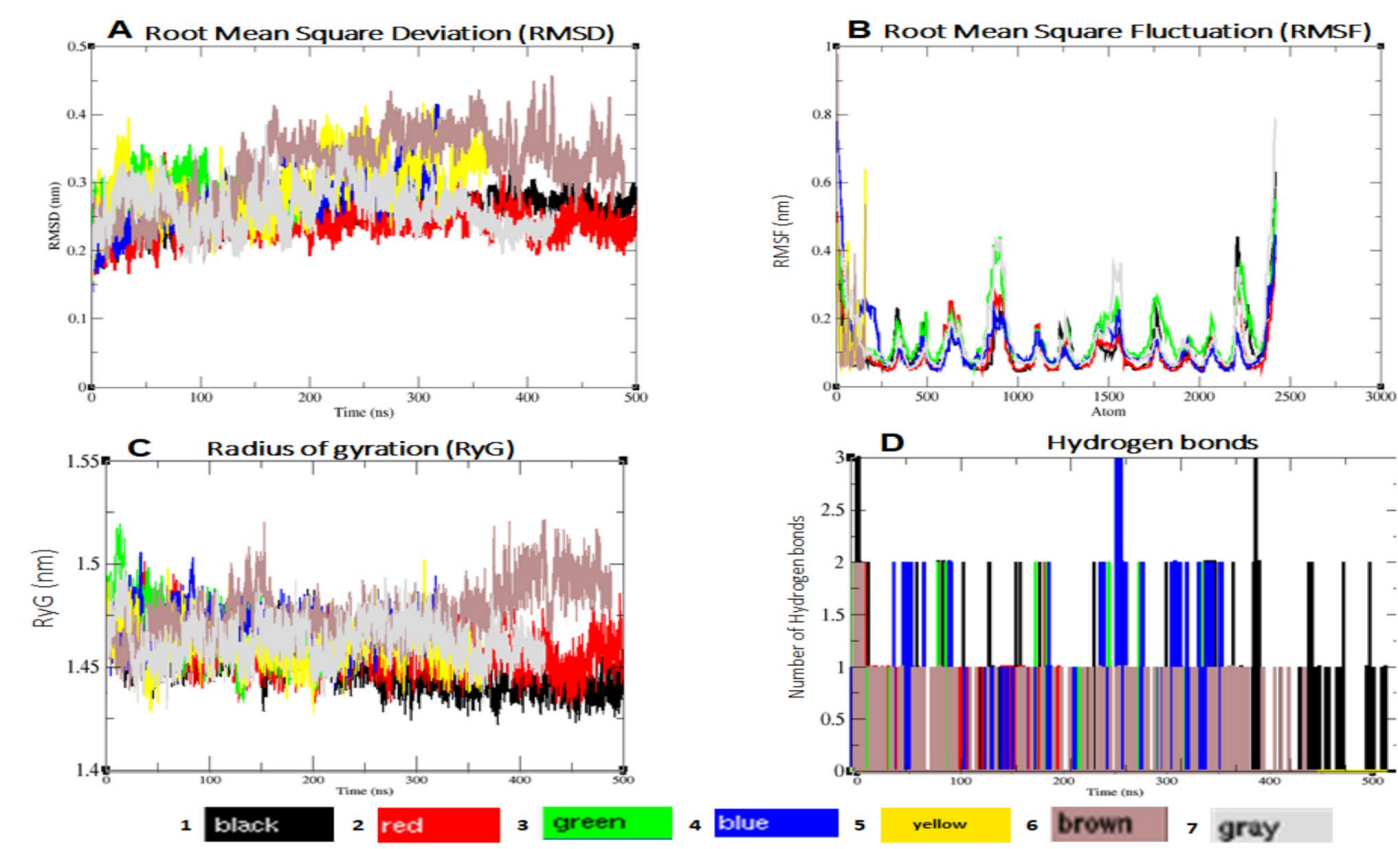
Trajectory analysis of the phytoconstituents and the 2KKI model complexes over a 500 ns simulation period. **A**: Root mean square fluctuations (RMSD), **B**: Fluctuations (RMSF), **C**: Radius of gyration (RyG) and **D**: Hydrogen bonds

The detailed analysis of the MD simulations for the 2KKI model (IL-1) and the phytoconstituents are summarised in Fig. 3 and Table 3.

**Table 3:** Molecular dynamics simulations analysis of the 2KKI model of IL-1 protein over 500 ns

Molecule/Compound identification (CID)	RMSD (nm)	RMSF (nm)	RyG (nm)	Hydrogen bonds	Non-bonded (kcal/mol)		
					Coulomb (C)	Leonnard-Jones (L-J)	Sum of C and L-J
Protein only	0.26 ± 0.02	0.11 ± 0.08	1.45 ± 0.01	–	–	–	–
Thalidomide	0.24 ± 0.02	0.11 ± 0.07	1.46 ± 0.00	3	− 7.36 ± 18.41	− 9.12 ± 16.27	− 16.48 ± 26.55
8122	0.28 ± 0.03	0.15 ± 0.08	1.47 ± 0.01	2	− 5.77 ± 11.42	− 76.47 ± 30.76	− 82.24 ± 21.09
33934	0.27 ± 0.04	0.12 ± 0.08	1.47 ± 0.01	2	− 13.72 ± 11.17	− 90.35 ± 52.92	− 104.07 ± 32.05
605626	0.29 ± 0.04	0.13 ± 0.09	1.46 ± 0.01	3	− 7.24 ± 13.96	− 27.49 ± 28.71	− 34.73 ± 21.34
638072	0.32 ± 0.05	0.13 ± 0.13	1.48 ± 0.01	0	1.13 ± 4.62	− 166.23 ± 47.40	− 165.10 ± 26.01
5363269	0.26 ± 0.03	0.14 ± 0.11	1.46 ± 0.01	2	− 6.02 ± 10.11	− 56.27 ± 35.93	− 62.29 ± 23.02

As shown in Table 3, the controls used for the 2KKI model were limited to the unbound protein (0.26  ± 0.02 nm) and thalidomide (0.24  ± 0.02 nm). The *Capparis sepiaria* phytoconstituents such as CID 5363269 (0.26  ± 0.03 nm), CID 33934 (0.27 ± 0.04 nm), CID 8122 (0.28  ± 0.03 nm), CID 605626 (0.29  ± 0.04 nm), and CID 638072 (0.32  ± 0.05 nm), showed similar RMSD values. T-tests showed no significant difference between thalidomide and CID 5363269 (*p* = 0.12), suggesting similar binding stability, while CID 638072 had a significantly higher RMSD (*p* < 0.05). These results indicate that certain phytoconstituents, particularly CID 5363269, are as effective as thalidomide for RA-related protein binding.

The RMSF values were consistent with the RMSD results, with the controls exhibiting lower RMSF values than the phytoconstituents. Thalidomide recorded the lowest RMSF at 0.11  ± 0.07 nm, closely followed by the unbound protein control at 0.11  ± 0.08 nm. Among the phytoconstituents, CID 33934 had the lowest RMSF at 0.12  ± 0.08 nm, followed by CID 605626 (0.13  ± 0.09 nm) and CID 638072 (0.13  ± 0.11 nm). CIDs 5,363,269 and 8122 showed slightly higher RMSF at 0.14  ± 0.11 nm and 0.15  ± 0.08 nm, respectively.

The RyG values, however, were remarkably uniform across both controls and phytoconstituents, spanning from 1.45  ± 0.01 to 1.48  ± 0.01 nm.

In terms of hydrogen bonding, both thalidomide and CID 605626 formed 3 hydrogen bonds, while CIDs 8122, 33934, and 5363269 each formed 2. Notably, CID 638072 did not engage in any hydrogen bonding.

For non-bonded interaction energies, all phytoconstituents exhibited higher binding affinities (i.e., lower potential energies) than thalidomide. CID 638072 led with the lowest potential energy at − 165.10  ± 26.01 kcal/mol, followed by CID 33934 (− 104.07  ± 32.05 kcal/mol), CID 8122 (− 82.24  ± 21.09 kcal/mol), CID 5363269 (− 62.29  ± 23.02 kcal/mol), and CID 605626 (− 34.73  ± 21.34 kcal/mol), with thalidomide having the least favourable energy at − 16.48  ± 26.55 kcal/mol.

#### Principal component analysis (PCA)

**Fig. 4:**
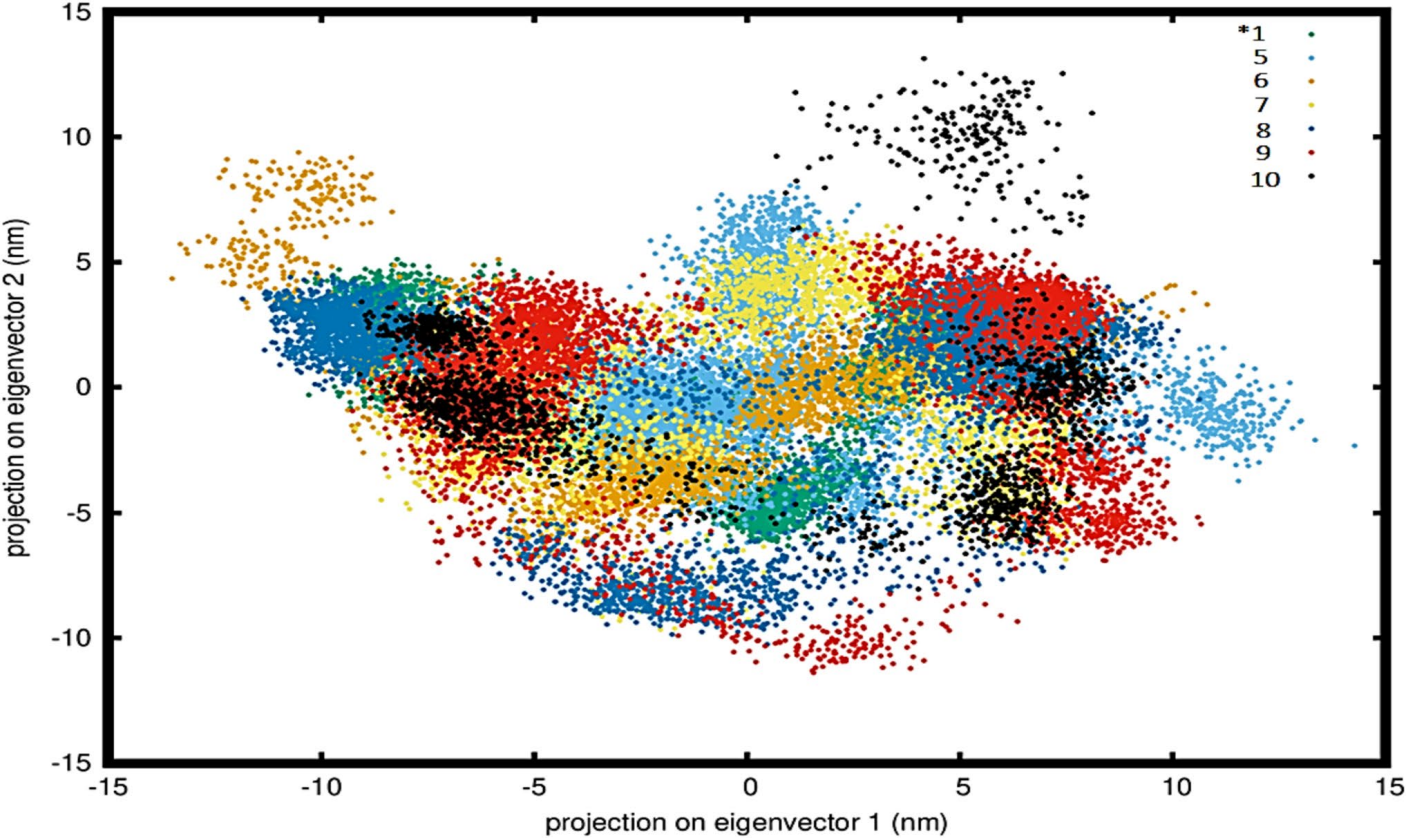
Principal component analysis (PCA) of model 2KKI bound to different phytoconstituents, over 500 ns. Unbound protein model is represented as *1 (2KKI model of IL-1) while the colorful phytoconstituents are depicted as 5: thalidomide, 6: CID 33934, 7: CID 605626, 8: CID 638072, 9: CID 5363269 and 10: CID 8122, respectively

The eigenvectors and eigenvalues of the covariance matrix were computed to identify the principal components, which were then plotted in 2D as illustrated in Fig. 4.

As shown in Fig. 4, similar to the trends seen in non-bonded interaction energies, all tested phytoconstituents demonstrated higher PCA % than the thalidomide control. Specifically, the PCA % recorded for CIDs 8122, 638072, 536329, 33934 and 605626 was 79%, 77.8%, 72.1%, 71.5% and 67.5%. Thalidomide, on the other hand, achieved a PCA % of 64.1%. Moreover, the unbound protein control exhibited a higher PCA % of 74.4%, surpassing most of the CIDs.

The controls in the 2KKI model also displayed higher PCA % percentages than those for the phytoconstituents. As shown in Fig. 4, all phytoconstituents occupied a more extensive subspace with less stable cluster conformations compared to the protein control. This suggests that these phytoconstituents possess fewer stable conformations.

#### Binding free energy calculations

The binding free energies for complexes involving the phytoconstituents with protein model 2KKI were calculated using the gmx_MMPBSA tool within GROMACS software. The results are detailed in Table 4, which also considers the components of the MM-PBSA, such as ∆G VDW, ∆G EEL, ∆G EGB, ∆G ESURF, and G TOTAL.

**Table 4:** MMPBSA analysis of the complex systems at the stable region, 100–200 ns (1001 frames), for the 2KKI model and the selected phytoconstituents

Energy component	*1	5	6	7	8	9	10
∆G VDW (kcal/mol)	–	− 1015.94 ± 17.01	− 972.64 ± 13.64	− 986.02 ± 12.71	− 1034.39 ± 12.92	− 1012.50 ± 13.93	− 995.29 ± 18.25
∆G EEL (kcal/mol)	–	− 990.14 ± 6.33	− 937.50 ± 7.43	− 957.20 ± 6.45	− 980.85 ± 7.42	− 933.91 ± 6.85	− 928.42 ± 5.94
∆G EGB (kcal/mol)	–	− 217.27 ± 4.61	− 257.55 ± 5.86	− 239.97 ± 4.51	− 213.59 ± 5.99	− 252.09 ± 5.68	− 251.35 ± 4.88
∆G ESURF (kcal/mol)	–	65.02 ± 1.13	71.46 ± 1.25	73.55 ± 1.22	64.42 ± 1.32	66.11 ± 1.09	66.64 ± 2.17
∆G TOTAL (kcal/mol)	–	− 2158.33 ± 7.27	− 2096.22 ± 5.99	− 2109.64 ± 6.22	− 2164.42 ± 6.91	− 2132.39 ± 6.89	− 2108.42 ± 7.81

*VDW* Van der Waals forces, *EEL* Electrostatic component of the internal energy, *EGB* polar contribution to solvation energy by GB/PB method

^*^1: 2KKI model of IL-1 protein; 5: thalidomide; 6: CID 33934; 7: CID 605626; 8: CID 638072; 9: CID 5363269 and 10: CID 8122, respectively

As shown in Table 4, CID 638072 had the highest binding free energy (∆G TOTAL) at − 2164.42  ± 6.91 kcal/mol, surpassing the control (thalidomide), and other phytoconstituents evaluated. Thalidomide followed at − 2158.33  ± 7.27 kcal/mol, with CID 5363269 at − 2132.39  ± 6.89 kcal/mol demonstrating the third highest binding free energy. The binding free energies for CIDs 605626, 8122, and 33934 were − 2109.63  ± 6.22 kcal/mol, − 2108.42  ± 7.81 kcal/mol, and − 2096.22  ± 5.99 kcal/mol, respectively. The binding energy was primarily driven by Van der Waals interactions (VDW) and the EEL.

## Discussions

### Protein and ligand selection and preparation

In molecular docking, it is necessary to optimise both the protein structure and to achieve their most stable conformational states. This optimisation renders them rigid, promoting the proper alignment of conformations into compact structures (Forli et al. 2016). This crucial step reduces potential errors in electrostatic interactions and mitigates the disturbances from hydrophobic interactions and conformational entropy, factors pivotal to binding affinity and specificity (Sousa et al. 2006). The process entails energy minimisation alongside conformational adjustments to eliminate steric clashes and unfavorable interactions, resulting in more reliable docking outcomes. As shown in Fig. 1, protein model 2KKI and ligands were effectively optimised, which in turn improved the docking accuracy and the prediction of binding interactions.

### Molecular docking calculations

As shown in Fig. 2, the control compound employed in this study exhibited binding energies ≥ − 6.0 kcal/mol, consistent with the study by Yende et al., (2021), who reported values above − 7.0 kcal/mol for their controls. A threshold of ≥ − 6.0 kcal/mol identified promising phytoconstituents (Dinesh et al. 2017; Kar et al. 2023; Sharma et al. 2023). The binding energy threshold ensured the identification of compounds with superior binding scores, thus highlighting the most promising candidates for further analysis.

The relationship between the number of amino acid residues involved in binding and the resultant binding energy was not straightforwardly proportional. This apparent discrepancy was also described in the study by Alberts et al. (2002) and they explained that this might be due to the molecular composition, specifically the presence of carbon and nitrogen atoms, and steric hindrances that limit atomic interactions, thus affecting potential bond angles and conformations (Alberts et al. 2002). Additionally, slight structural modifications in the target protein could either enhance or inhibit the binding of phytoconstituents to active sites, thereby affecting binding energies (Alberts et al. 2002). Despite these variations, the control compounds demonstrated the highest binding scores, consistent with the study by Yende et al., (2021). Three independent studies explained that this could potentially be due to their specific orientations within the binding pockets (Kalita et al. 2020) and the grid box settings used in the molecular docking process (Halgren et al. 2004; Morris et al. 2009). Furthermore, the controls in this study exhibited higher binding scores, compared to a study by Morris et al. (2009), likely due to differences in docking algorithms (Morris et al. 2009). The current study utilised AutoDock VinaXB, which incorporates both the AutoDock Vina algorithm and an innovative empirical halogen bond scoring function (Koebel et al. 2016), while Morris et al. (2009) employed AutoDock4 and the graphical user interface AutoDockTools, with limited flexibility in the receptor (Morris et al. 2009). Our findings also align with the strong binding affinities Bai et al. (2021) and Zia et al. (2020) reported for their studies on TNF-α inhibitors (Bai et al. 2021; Zia et al. 2020). However, the interactions of the compounds we selected for interaction studies with IL-1, show unique residue patterns, suggesting a distinct potential of *C. sepiaria* and its derived phytoconstituents for the treatment of RA.

Prior to conducting ADMET and MD simulations, all phytoconstituents meeting the binding energy cut-offs were evaluated using PLIP, as summarised in Table 1. As shown in Table 1, the selected phytoconstituents exhibited common active amino acid residues, contributing to their similar binding energies. Common active amino acid residues include Asp65, Phe12, Thr123 and Pro128. Also, additional residues bound via hydrogen bonding including Val125, Ser124, Gly145, Lys63 etc. This pattern is consistent with findings by Wadanambi and Mannapperuma (2021) where inhibitors with shared chemical properties demonstrated analogous biochemical interactions with their protein targets (Wadanambi and Mannapperuma 2021). Furthermore, the observation that these phytoconstituents bind to nearly identical active residues suggests they might interact with the epitope of 2KKI (Mohan and Yu 2011), indicating their potential as therapeutic agents for conditions like arthritis.

### ADMET studies

Table 2 outlined the application of Lipinski's rule of five, which provides a framework for assessing the drug-likeness of phytoconstituents, with emphasis on their solubility in water, oral bioavailability, and intestinal permeability (Ojo et al. 2022). While adherence to these rules is not absolute for all phytoconstituents, and their pharmacological effectiveness does not always directly correlate with compliance with Lipinski’s criteria, significant deviations can impede the achievement of optimal oral activity (Lipinski 2004). Similarly, violations of these rules may also present challenges associated with oral activities of these phytoconstituents (Adekiya et al. 2022; Fadaka et al. 2021).

As shown in Table 2, the selected phytoconstituents were in line with the oral druggability and bioavailability as outlined by Lipinski’s rule of five. These rules, together with Veber’s rule, indicate that compounds with hydrogen bond acceptors and donors numbering 12 or fewer possess enhanced oral bioavailability (Veber et al. 2002), were pivotal in the selection of phytoconstituents. Consequently, the phytoconstituents highlighted in Table 2 largely satisfied the criteria necessary to be regarded as prospective candidates for orally administered drugs.

## MD simulations

Figure 3 provided a summary of the MD simulation analysis for the selected phytoconstituents and the 2KKI model of IL-1. The 500 ns MD simulations were deemed sufficiently robust, as previous studies have typically used trajectories shorter than 200 ns trajectories, while also incorporating relevant physiological and physicochemical data (Abdullah et al. 2023; Andalib et al. 2023). The MD simulation analysis are also summarised in Table 3.

As shown in Table 3, the control compound (thalidomide) showed superior performance in terms of RMSD (Fig. 3A) and RMSF (Fig. 3B) compared to the phytoconstituents. The higher RMSF values may be associated with the flexibility of loop regions and alpha helices (Fig. 3B). A comparable study observed significant fluctuations throughout the simulation, with peak RMSF values ranging from 0.640 to 6.48 nm for the 4OVZ-I-Asarinininin complex (Lakhera et al. 2021). Despite these fluctuations, all phytoconstituents displayed higher binding scores than thalidomide, possibly due to the presence of water molecules and ions in the simulation environment (Brooks et al. 2009; Yan and Levy 2018). Another study by Martin et al. (2023) documented higher potential energies for tested compounds relative to the positive control (Martin et al. 2023), consistent with our observation of higher binding scores but less stable RMSD/RMSF profiles. The elevated potential energies may reflect additional interactions formed by phytoconstituents like CID 33934. However, these interactions did not translate to lower RMSD, possibly due to the flexibility binding pocket of IL-1. Future work will optimise phytoconstituents to reduce RMSF and enhance stability, potentially through chemical modifications guided by analysis using LigPlot software.

In the PCA analysis, the phytoconstituents covered a wider conformational space with less stable cluster formations compared to the protein only control, suggesting lower conformational stability. This resulted in increased flexibility (higher RMSF) in loop regions and a reduction in overall PCA %. The PCA data revealed that clusters were well-defined, occupying a minimum subspace on the protein. Applying PCA to a protein trajectory is reflected as “Essential Dynamics”, which extracts essential motion required for model stabilisation (David and Jacobs 2014). During MD simulations, density distribution analysis of the molecular coordinates of the 2KKI model is an essential step to evaluate both the spatial atomic density and changes in atomic orientation over time. This analysis provides insights into the structural stability and conformational preferences of biomolecular systems under simulated physiological conditions. A comparable approach was utilised by Singh and Singh (2024), who identified a stable density region associated with their model protein. This region corresponded to a low-energy conformation, indicating a thermodynamically favorable state that contributed to the structural integrity of the system (Singh and Singh 2024).

For the MMPBSA analysis involving the 2KKI model, compound CID 638072 exhibited the highest binding free energy among all compounds tested, outperforming even the control, thalidomide. This superior binding affinity is likely due to more stable and energetically favorable interactions within the binding pocket of the target protein, thereby suggesting a stronger and more persistent complex formation (Maszota-Zieleniak et al. 2021). The total binding free energy for CID 638072 was primarily driven by VDW and EEL energies, which attributed to the overall interaction strength. This observation aligns with the findings of Oo et al. (2016), who also reported VDW and EEL terms as key contributors to binding stability in similar molecular systems (Oo et al. 2016), Moreover, the MMPBSA results were in strong agreement with molecular docking data, further validating the reliability of CID 638072 as a high-affinity compound and reinforcing the consistency between dynamic binding simulations and static docking predictions.

Compound CID 638072 is well-documented for its anti-inflammatory properties, although its precise molecular mechanisms remain incompletely characterised (Huang et al. 2009; Kim and Karadeniz 2012; Sánchez-Quesada et al. 2018). A study by Cárdeno et al. (2015) demonstrated that squalene exerts significant anti-inflammatory effects by modulating key inflammatory mediators, suggesting potential therapeutic relevance in RA (Cárdeno et al. 2015). Similarly, CID 33934, identified in various plant extracts, has been associated with immunomodulatory, antipruritic, and antihypoxic effects (Hamsalakshmi et al. 2021; Sharmila et al. 2024). Although no direct immunomodulatory data are available for CID 605626, structurally similar phytoconstituents, such as curcumin derivatives, including fluorinated analogues like Shiga Y6, are known to downregulate pro-inflammatory cytokines (e.g., TNF-α, IL-6) and inhibit key signaling pathways (e.g., NF-κB, MAPK), highlighting the potential of CID 605626 to exert comparable effects (Aldoghachi et al. 2024). CID 5363269 has demonstrated strong antioxidant and anti-inflammatory properties, especially in nanoemulsion formulations, where it enhances bioavailability and protects cells from oxidative stress (Zhang et al. 2016). Finally, CID 8122 has been shown to suppress the reticuloendothelial system, leading to a 60–70% reduction in B-cell antibody responses to polysaccharide antigens, thereby modulating immune tolerance through the inhibition of macrophage-mediated humoral responses (Huang et al. 2023).

## Conclusion

An extensive assessment of diverse phytochemicals, which have previously been shown to be present in *C. sepiaria,* was undertaken to determine their efficacy in inhibiting IL-1 (using the 2KKI model) for RA therapy. IL-1 is a is a pro-inflammatory cytokine and a validated therapeutic target in the treatment of RA, where its inhibition is associated with a significant anti-inflammatory response and disease modulation. Molecular docking analysis using AutoDock VinaXB revealed five promising phytoconstituents including CID 8122, CID 33934, CID 605626, CID 638072, and CID 5363269, which met or exceeded the binding energy threshold (≥  − 6.0 kcal/mol). Notably, CID 638072 (squalene) demonstrated the most favorable binding score (− 7.0 kcal/mol) and the lowest binding free energy (− 2164.42  ± 6.91 kcal/mol), outperforming both the control compound (thalidomide) and other tested phytoconstituents. Squalene is well-documented for its anti-inflammatory properties, although its precise molecular mechanisms remain incompletely characterised. While our study did not initially elaborate on this, the current findings support further investigation into immunomodulatory role of squalene, and reinforce its promise as a candidate for RA drug development. Similarly, the flavonoids evaluated in this research possess many biological properties, including anti-inflammatory, antibacterial, analgesic, antitumor, hepatoprotective, antioxidant, and antidiabetic effects. These results suggest new research pathways for utilising these phytoconstituents in drug design efforts to counteract IL-1 for RA.

In addition, this study highlights the pivotal role of computational techniques in the initial stages of drug discovery, serving as a cost-effective, time-efficient, and high-throughput approach to screen, evaluate, and predict the bioactivity of numerous compounds prior to in vitro and in vivo testing. Through molecular docking, molecular dynamics simulations, and free energy calculations such as MMPBSA, researchers can gain valuable insights into the binding affinities, stability, and interaction profiles of candidate compounds with target proteins. These computational strategies not only accelerate the identification of promising lead compounds but also help in understanding molecular mechanisms of action, guiding experimental validation, and reducing the overall risk and expense associated with traditional drug development pipelines. Furthermore, applying these methods to study phytoconstituents that has been identified in plants that has been used for millennia in traditional medicine can assist to validate the traditional medicinal uses of these plants. These methods allow for the rapid and accurate prediction of molecular interactions and binding affinities, thus accelerating the discovery of potential therapeutic candidates. The integration of such computational insights with subsequent in vitro and in vivo experiments will be essential to ascertain the effectiveness and safety profiles of these identified compounds. In addition, exploration of additional IL-1 crystal structures, to investigate their synergistic effects of phytoconstituents should be pursued.

### Limitations

This study relied on in silico methods, and lacking in vitro and in vivo validation. Phytochemical composition may vary due to environmental factors, and the focus on 2KKI may not capture all IL-1 conformational dynamics. Future studies should address these gaps to confirm therapeutic efficacy of *C. sepiaria* for RA.

### Future perspectives

Further studies should be conducted because the data collected here are purely related to computational analyses and need to be tested experimentally in vitro and in vivo to prove these hypotheses.

Future research should also include validation of binding efficacy, toxicity, and therapeutic potential, explore additional IL-1 crystal structures, and investigate synergistic effects of phytoconstituents.

## Data Availability

No datasets were generated or analysed during the current study.
